# Subcellular Transcriptomics and Proteomics: A Comparative Methods Review

**DOI:** 10.1016/j.mcpro.2021.100186

**Published:** 2021-12-16

**Authors:** Josie A. Christopher, Aikaterini Geladaki, Charlotte S. Dawson, Owen L. Vennard, Kathryn S. Lilley

**Affiliations:** 1 Department of Biochemistry, Cambridge Centre for Proteomics, University of Cambridge, Cambridge, UK; 2 Milner Therapeutics Institute, Jeffrey Cheah Biomedical Centre, Cambridge, UK; 3 Department of Genetics, University of Cambridge, Cambridge, UK

**Keywords:** spatial proteomics, spatial transcriptomics, imaging, proximity labeling, cellular fractionation, BAP, biotin acceptor peptide, bDNA, branched DNA, cyTOF, cytometry by time of flight, DFHBI, 3,5-difluoro-4-hydroxybenzylideneimidazolidinone, ER, endoplasmic reticulum, EV, extracellular vesicle, FFE, free-flow electrophoresis, FIFFF, flow field-flow fractionation, FISSEQ, fluorescent *in situ* sequencing, FP, fluorescent protein, HPA, Human Protein Atlas, IFC, imaging flow cytometry, IMC, imaging mass cytometry, LOPIT, localization of organelle proteins by isotope tagging, MCP, bacteriophage MS2 coat protein, MERFISH, multiplexed error-robust FISH, MS, mass spectrometry, MSI, MS imaging, PAINT, point accumulation in nanoscale topography, PCP, P77 bacteriophage coat protein, PM, plasma membrane, PTM, post-translational modification, RBP, RNA-binding protein, RIP, RNA-co-immunoprecipitation, scRNA-Seq, single-cell RNA-Seq, seqFISH, sequential barcoding FISH, SINC-Seq, single-cell integrated nucRNA and cytRNA sequencing, TREAT, 3(three)′-RNA end accumulation during turnover, TRICK, translating RNA imaging by coat protein knockoff

## Abstract

The internal environment of cells is molecularly crowded, which requires spatial organization *via* subcellular compartmentalization. These compartments harbor specific conditions for molecules to perform their biological functions, such as coordination of the cell cycle, cell survival, and growth. This compartmentalization is also not static, with molecules trafficking between these subcellular neighborhoods to carry out their functions. For example, some biomolecules are multifunctional, requiring an environment with differing conditions or interacting partners, and others traffic to export such molecules. Aberrant localization of proteins or RNA species has been linked to many pathological conditions, such as neurological, cancer, and pulmonary diseases. Differential expression studies in transcriptomics and proteomics are relatively common, but the majority have overlooked the importance of subcellular information. In addition, subcellular transcriptomics and proteomics data do not always colocate because of the biochemical processes that occur during and after translation, highlighting the complementary nature of these fields. In this review, we discuss and directly compare the current methods in spatial proteomics and transcriptomics, which include sequencing- and imaging-based strategies, to give the reader an overview of the current tools available. We also discuss current limitations of these strategies as well as future developments in the field of spatial -omics.

## Highlights

• Subcellular information of protein and RNA give insights into molecular function.
• This review discusses strategies available to measure subcellular information.
• Hybridization of methods shows promise for exploring the composition of organelles.
• Advances are aiding understanding of the organisation and dynamics of cells.

## Graphical Abstract

**Figure:**
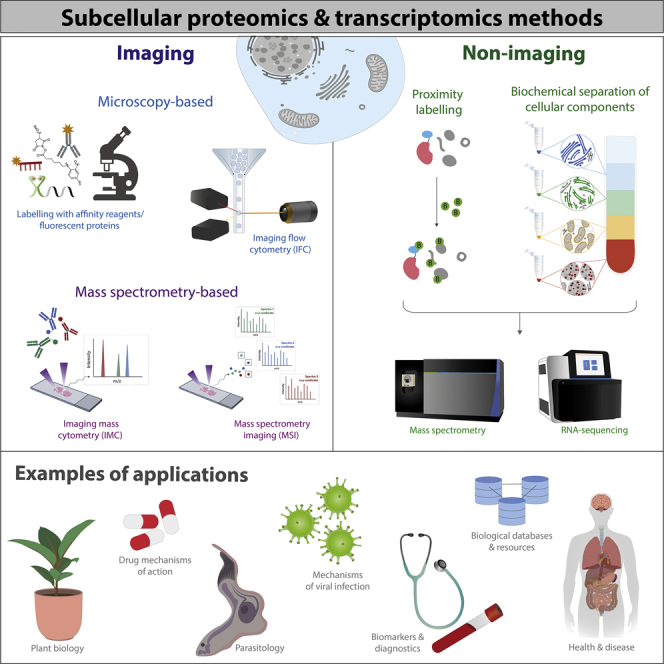

## In Brief

The interior of the cell is molecularly crowded. Its compartmentalization within organelles enables the regulation of biochemical processes and allows multifunctionality of proteins and RNAs. Subcellular information can thus give insights into the function of these biomolecules. Multiple techniques to measure such information have been established, with ever-increasing throughput and sensitivity. These techniques are covered in this review, and demonstrating their application is providing valuable insights into cellular biology, such as aiding our understanding of single-cell heterogeneity and posttranslational modifications.

Molecular biology is the study of cellular functions *via* processes such as molecular synthesis, modification, and interactions. RNA and proteins can have multiple roles and interacting partners that require close physical proximity to each other within the cell to function. Therefore, precise control of localization or colocalization by selective congregation and isolation of biochemical processes is integral and intrinsically linked to cellular functions. For instance, in context of transcription and translation, mRNA is shuttled out of the nucleus, where it docks at ribosomes within the cytosol, at the endoplasmic reticulum (ER) or near the mitochondria, dependent on the coded protein and cellular conditions (1, 2, 3). Translation of mRNA at the coded protein's functional site, rather than at a singular canonical and/or punctate location, is clearly demonstrated within polarized cells, such as neurons or intestinal epithelial cells (4, 5). Hence, studying subcellular localization not only gives insights into the organization of cellular compartments but how cells function; so techniques that provide spatial context are important tools in molecular biology.

The relationship between DNA, RNA and proteins does not represent a linear dogma. Interactions, or “interactomes,” between nucleic acids and proteins are fundamental for cellular function. RNA-binding proteins (RBPs), originally thought to exclusively function in gene regulation *via* ribonucleoprotein complex formation, have now been shown to have more extensive interplay between protein and RNA interactomes (6). A prime example of RNA-mediated and RBP-mediated regulation *via* subcellular relocalization is the short noncoding RNA transcript Y3 RNA, which orchestrates translocation of the RBP Rho 60-kDa autoantigen between the cytosol and nucleus as part of a UV-induced survival mechanism (7, 8). A more classic example of subcellular control is during the cell cycle, where cyclins and cyclin-dependent kinases traffic between nuclei and cytosol (9). An in-depth immunofluorescence study has recently captured single-cell variability of subcellular composition during the cell cycle (10).

Aberrant trafficking of RNA and protein has been implicated in several pathological conditions, including amyotrophic lateral sclerosis and pulmonary atrial hypertension, respectively (11, 12). A well-documented example of mislocalization causing severe disease is the most common mutation in cystic fibrosis, F508del. Immunofluorescence and subcellular fractionation strategies have shown that this mutation causes the cystic fibrosis transmembrane regulator ion channel to misfold and accumulate at the ER, preventing cystic fibrosis transmembrane regulator expression at the plasma membrane (PM) and, consequently, impairing mucus clearance in the lungs (13, 14, 15). This has aided the design of pharmacological intervention to correct this misfolding and subsequent mislocalization (16). In many of these cases, early stages of disease can be identified by translocation events, which can precede or be independent to detectable changes in gene expression and, therefore, can only be studied at the subcellular level (17). Despite this, temporal or differential expression is more commonly studied because it is more straightforward, though novel tools to study the spatial dimension on an -omics scale are opening new opportunities for a better understanding of cellular function.

Spatial proteomics and transcriptomics have often been reviewed independently with technical details covered in previous articles (18, 19, 20). Here, we outline and directly compare methods (summarized in Table 1) that interrogate the spatial transcriptomic and proteomic within subcellular compartments of cells, rather than spatial information at the tissue level, and suggest gaps in technology in need of further advancement. We aim to provide a resource for newcomers to spatial -omics who wish to unpick the busy, yet spatially organized, environment within cells.

**Table 1:** Summary of each method covered within this review

Method	Principle	Examples of biological insights	Live, fixed, or lysed samples?	*In situ*?	Targeted?
Imaging					
Affinity reagents	Exogenous dyes or probes (*e.g.*, antibodies or oligonucleotides) designed to target specific molecules of interest (MOIs)	The largest database of human protein subcellular localizations using stringently validated antibodies, giving insights into cell variability and mapping subcellular localization of SARS-coronavirus 2 interactors (37, 52). smFISH aided the understanding of how liquid–liquid phase separation aids formation of rotavirus replication factories (considered virus-made membraneless organelles) (340)	Primarily fixed samples (exception of live FISH)	✓	Targeted, label MOI
Fluorescently tagged proteins	Fluorescent proteins (typically) genetically fused to MOI and, therefore coexpressed with the MOI	Genetically fused fluorescent proteins were used to gain insight into the pH- and receptor-dependent endocytic entry of severe acute respiratory syndrome virus into the host cell (92)	Live/fixed	✓	Targeted, label MOI
IFC	A combination of flow cytometry and microscopy to capture spatial information using fluorescent probes	An IFC method was developed to provide a more informative diagnostic tool for types of acute leukaemia (161)	Live/fixed	✓	Targeted, label MOI
IMC	Uses heavy-metal probes conjugated to antibodies, which ablated pixel by pixel and measured using MS. This improved multiplexing of probes because of the reduced spectral overlap compared with fluorescent strategies	Used for cellular phenotyping of breast cancer and lesions in multiple sclerosis and lymphoid organs (166, 171, 172). Primarily used for tissue-level insights, rather than subcellular, though some subcellular information is achievable with the method	Fixed	✓	Targeted, label MOI
MSI	Similar to IMC, but ablation leads to ionization of all molecules within the pixel, producing a separate spectra per pixel of the sample	Primarily, still tissue-level resolution, rather than subcellular resolution. Has been used for intraoperative imaging of pituitary adenomas for biomarkers that are usually difficult to detect efficiently (177)	Typically fixed	✓	Untargeted, cell-wide
Biochemical separation					
Basic centrifugation/detergent based	Uses targeted centrifugation or detergent step(s) to achieve enrichment of a specific cellular component or organelle of interest	Used in the study of mitochondrial transport in *Trypanosoma brucei* to aid understanding of parasitic physiology (219) and gain insights into proinflammatory gene regulation in context of subcellular dynamics of macrophages from mice (226)	Lysed, *in vitro*		Untargeted, enrich organelle(s) of interest
Correlation profiling	Uses multiple centrifugation or detergent steps of increasing spin speed/time or solubility, respectively, to collect an abundance profile of one or multiple subcellular components. Can be used for cell-wide analysis of molecules	Used to track the subcellular proteome of host cells over the course of human cytomegalovirus infection in a spatial and temporal context (237). Also, used to identify that lysosomal trapping is important for the efficacy of drugs that aid antigen presentation (259)	Lysed, *in vitro*		Untargeted, cell-wide
Electrophoresis based	Separates subcellular components *via* their charge state using modified electrophoresis techniques	Used to assess the protein composition of the secretory pathway in plants that are otherwise difficult to resolve with centrifugation because of their similar density (359)	Lysed, *in vitro*		Untargeted, cell-wide
Proximity labeling					
BioID and APEX	Fusion of bait protein(s) to either a biotin ligase (*e.g.*, BioID) or peroxidase (*e.g.*, APEX) that covalently labels molecules in immediate proximity of the bait with a small and exogenous substrate. The substrate can then be purified along with the labeled molecules	BioID has revealed novel organellar components of the *Trypanosoma brucei*, flies, and worms (295, 302, 304, 308) and identifying novel proteins involved in hyperpolarization that are linked to neurodegenerative diseases (314, 315) APEX-Seq identified stress type–dependent RNA interactions with stress granules (18, 331)	Lysed, *in vivo* labeling		Untargeted, label organelle(s) of interest

The table includes a short description about the principle of each method, examples of their biological insights or applications, and basic comparisons of the characteristics of the methods.

## Imaging the Spatial Transcriptome and Proteome

### Microscopy-Based Imaging

Microscopy is the most well-established and largest branch of imaging with a variety of labeling strategies for targeting proteins and transcripts, often at a single-cell level. Conducting microscopy studies on a global spatial scale can be challenging and laborious because of costly generation of antibodies or recombinant organisms and limited multiplexing capacity. In addition, sample preparation is rarely a one-size-fits-all process. For example, fixing is usually dependent on the subcellular compartment of interest, and phototoxicity is a limiting factor in live-cell imaging. Fixing cells can disrupt molecular organization and macroorganization and structures, causing artificial localization of molecules (21) but does not suffer from issues of phototoxicity and can capture snapshots of transcripts and proteins, which rapidly fluctuate or have low copy number. Some applications are limited to fixed samples only, such as immunofluorescence or FISH, whereas others have the capacity for live-cell imaging, such as genetically fusing fluorescent tags. Recent emergence of high-throughput and super-resolution microscopy has allowed mid-scale to large-scale spatial studies of transcripts and proteins, permitting quantitative measurements alongside the “seeing is believing” aspect at which imaging excels. Furthermore, while simultaneous genome-wide live-cell imaging is not yet possible, recent advancements in the field of high-content imaging are enabling faster image acquisition at higher resolution, though often with a trade-off between the two (22). Both the technological advancements of the instrumentation and bioimaging informatics have been extensively reviewed (23, 24, 25, 26, 27).

Here, we briefly discuss the main labeling options and some alternative imaging approaches, whilst outlining the advantages and disadvantages, and giving representative examples of their use in subcellular research, specifically in the context of large-scale spatial studies. The following labeling strategies are not necessarily exclusive to each other, and combinational labeling protocols have been documented (28, 29, 30, 31, 32).

#### Visualization of Using Affinity Reagents

##### Antibodies and Organelle-Specific Dyes

**Fig. 1:**
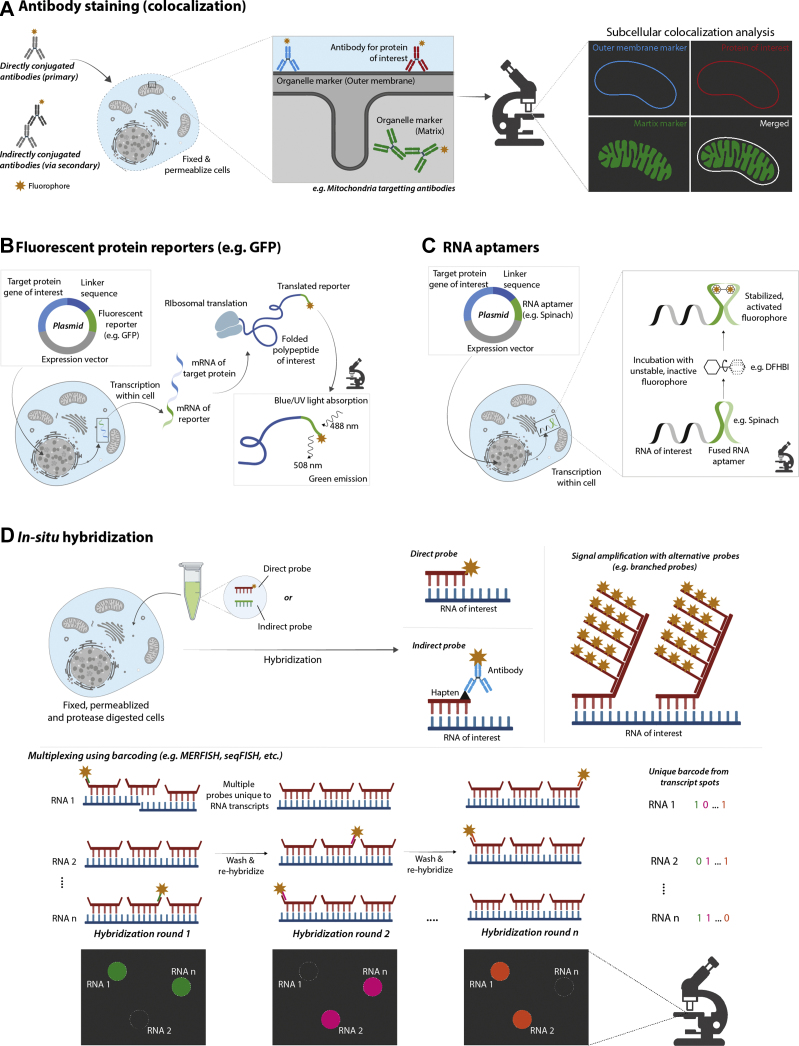
**Microscopy-based imaging approaches for subcellular proteomics or transcriptomics, focusing on the probing strategies.***A*, traditional antibody staining involves probing subcellular targets (such as the mitochondrial substructure) using monoclonal antibodies. These may be directly conjugated to a fluorescent label (direct immunofluorescence) or with a fluorescently labeled secondary antibody (indirect immunofluorescence). To determine subcellular location of proteins, an antibody against an organelle marker or a dye must be used alongside an antibody against the protein of interest. Then analysis can be performed to determine and quantify the colocalization of these antibodies/dyes. *B*, fluorescent protein reporters, such as GFP, can be genetically engineered to be fused and expressed with a target gene/protein of interest. Therefore, allowing confocal imaging of molecules that have no antibody or require live-cell imaging. In MS2 labeling systems for RNA, fluorescent reporter proteins can be genetically fused to MCP. *C*, RNA aptamers are an alternative to MS2 systems for labeling RNA, which allow for fusion of an RNA structure that binds and stabilizes an exogenous fluorescent molecule (*e.g.*, DFHBI). RNA aptamers can be used either as affinity reagents or as reporters. *D*, *in situ* hybridization (ISH) employs a variety of antisense nucleic acid probes for the detection of RNA of interest in permeabilized and fixed cellular material. Recent ISH strategies have allowed for highly multiplexed experimental designs using molecular barcoding (*e.g.*, seqFISH and MERFISH). DFHBI, 3,5-difluoro-4-hydroxybenzylideneimidazolidinone; MCP, bacteriophage MS2 coat protein; MERFISH, multiplexed error-robust FISH; seqFISH, sequential barcoding FISH.

In the case of proteins, the use of antibodies against specific endogenous proteins of interest is often known as immunofluorescence or immunocytometry. Immunofluorescence can be highly sensitive when using signal-amplifying reagents, such as secondary antibodies conjugated to various fluorophores. Readily available commercial antibodies make comparative studies of protein localization in different cell or tissue samples easy and fast, particularly in commonly used model organisms, such as humans and mice. Finding commercial antibodies for some less well-studied species and proteins can be more difficult. This can be overcome by genetically fusing an epitope, such as FLAG, to the protein of interest and then using an antibody against this epitope to indirectly label the protein. However, in this case, a fluorescent protein (FP), such as GFP, genetically fused to the protein is often favored as it negates the need for the antibody-labeling step. Chemical organelle–specific dyes, such as 4′,6-diamidino-2-phenylindole for nuclei staining, can also be used alongside antibodies. Reviews are available detailing such dyes (33, 34). It should be noted that antibodies are prone to batch-to-batch variability and poor specificity that can yield false results from nonspecific and variable binding. These drawbacks have caused major reproducibility crises amongst the scientific community (35). In recent years, however, there has been a huge drive to address this key issue with commercial suppliers providing extensive validation and moving toward recombinant products with less batch variability. In addition, with the increasing accessibility of CRISPR technology, validating specificity of antibodies using CRISPR knockouts is becoming common practice. Immunofluorescence-based methods are also restricted to static end-point measurements since such experiments require cell fixation and permeabilization prior to intracellular staining (Fig. 1*A*). Sample preparation can be very context specific, and inappropriate selection of fixation and permeabilization approaches can affect protein localization by introducing artifacts or causing loss of soluble proteins (20, 36). However, standardization of sample preparation and developments in automation has allowed multiplexing of off-the-shelf antibodies to improve throughput (37, 38).

Limited global spatial proteomics experiments have been conducted because of the aforementioned restrictions. The largest immunofluorescence-based subcellular proteomics study performed to date is the work of the Cell Atlas database. This work is part of the wider Human Protein Atlas (HPA) initiative, aiming to document the entirety of the human subcellular proteome in different human cell and tissue types to elucidate protein function and create a comprehensive biological resource for human proteins in health and disease (39, 40, 41). HPA has collaborated with other international-scale projects, such as UniProt, NextProt, GO, ELIXIR, to provide publicly available databases of subcellular information for the wider scientific community (42, 43, 44, 45). During the past two decades, a near proteome-wide collection of antibodies has been created and validated for the purpose of this initiative (46, 47, 48, 49, 50). This work used 14,000 antibodies to systematically map the spatial distribution of 12,003 proteins at single-cell resolution to one or more of 30 different subcellular niches. Of those proteins, 5,662 lacked subcellular localization information in the literature prior to this study. This classification was performed using a combination of manual and computational image analysis approaches (40, 51). Notably, the images were obtained using high-resolution confocal microscopy, enabling assignment of proteins to fine and less-well characterized cellular structures, such as microtubule ends, cytokinetic bridge subcompartments, and the nucleolar fibrillar center, as well as to functionally uncharacterized subcellular niches, such as rods and rings. Moreover, this work showed that approximately half of all human proteins (6,163 out of 12,003 proteins in this dataset) localize to multiple (two or more) subcellular niches. This dataset also revealed that more than one-sixth of the human proteome displays variability in terms of expression levels or subcellular distribution at the level of single cells (40). During the coronavirus disease 2019 pandemic, with collaborators, HPA turned to mapping the distribution of the virus' key host interactor, ACE2, across >150 human tissues, as well as the human interactome of coronavirus disease 2019 with the aim to determine whether readily available drugs can be repurposed in the fight against the virus (37, 52).

In immunofluorescence, the number of proteins of interest that can be probed in one sample is largely limited to the number of fluorochromes that can be used without causing signal interference by spectral overlap or fluorescent bleed-through into other channels. Therefore, traditionally, only around 4 to 6 fluorochromes could be used at a time, where each primary antibody is labeled with its unique fluorochrome (*e.g.*, conjugated to a secondary antibody). Recent developments in using either cyclical probing with antibodies, such as cyclic immunofluorescence, or using them in combination with other types of probes, such as oligonucleotides as molecular barcodes in co-detection by indexing, has allowed for improved multiplexing (38, 53, 54, 55). Another example of overcoming fluorescent signal overlap was the use of unique and fidentifiable DNA origami structures with the blinking kinetics of DNA-point accumulation in nanoscale topography (DNA-PAINT) that allowed multiplexing antibody probes in a single channel with super-resolution microscopy (56, 57).

The targeting of RNA transcripts directly *via* antibodies is far less prevalent than proteins. Antibodies against RNA antigens do exist; however, these are limited to global RNA applications. For example, antibodies against a subtype of RNA, such as rRNA, or for epigenetic applications, such as certain global modifications of RNA (*e.g.*, methylation or acetylation groups). This restricts their primary use to immunoblotting or immunopurifications and is not typically applicable to imaging (58, 59).

##### *In Situ* Hybridization

*In situ* hybridization was first discovered as a useful nucleic acid labeling tool in 1969 using radioactive tritium-labeled antisense sequences to image the nuclei of frog eggs (60). FISH was soon adopted as a safer and more stable alternative (61). The oligonucleotides used in FISH are designed to hybridize on the RNA target by sequence complementarity. These oligonucleotides are labeled either directly or indirectly, *via* a secondary probe (such as an antibody) conjugated to a fluorophore (Fig. 1*D*). Single-molecule resolution was enabled by “tiling” multiple antisense probes along a sequence of interest to boost signal and has been a powerful tool in understanding the role of RNA localization in biology, such as in meiosis and neuromuscular junctions (62, 63, 64). The main restriction of FISH has been its low throughput and need to be performed in fixed cells to prevent RNase and DNase degradation of nucleic acids, limiting its use for temporal applications. However, FISH imaging in live cells, known as “live FISH,” has been achieved with the caveats of using toxic permeabilization techniques and rapid sequestering of the molecular beacons in the nucleus (65, 66). It has only been with the recent developments within CRISPR–Cas9 technology that live FISH has been possible without such drawbacks (67). Despite live-imaging alternatives, such as aptamers, RNA FISH is still a gold-standard technique for RNA localization, and recent advancements, such as CRISPR–Cas9, have kept it current and pervasive.

FISH has a multitude of available signal-amplifying probes to choose from, which are particularly useful for overcoming hurdles commonly found in difficult targets and samples, such as short noncoding RNA and tissues (68). Generally, these probes have branched structures that increase the molecular surface area for multiple fluorophores to bind to the molecule of interest, which form the basis for single-molecule inexpensive FISH, FISH with sequential tethered and intertwined oligodeoxynucleotide complexes, branched DNA (bDNA) FISH, and hybridization chain reaction FISH (69, 70, 71, 72). For targets that require particularly high specificity, such as short noncoding RNAs, padlock probes can covalently “lock” and amplify the signal using a rolling circle mechanism (73, 74). bDNA probes were used for a large-scale imaging study, which targeted 928 genes involved in cancer, endocytosis, and metabolism at a single-cell level (75). The use of enzyme amplification of *in situ* hybridization probes and 96-well plates enabled mapping of mRNA dynamics in embryogenesis of *Drosophila*, achieving analysis of 3,370 transcripts and demonstrated a correlation between mRNA localization and subsequent protein localization and function (76). In addition, super-resolution FISH was used alongside RNA-Seq methods to track the dynamics of proteins in dendritic cells (4).

Multiplexing is also a powerful feature of FISH with easy to perform probe generation and sequential rehybridization of the sample, allowing for multiple rounds of reprobing and fluorescent barcoding of thousands of molecules, with minimal loss of signal (77). Novel methods, such as multiplexed error-robust FISH (MERFISH) and sequential barcoding FISH (seqFISH), exploit such characteristics and, in theory, have the capability of generating spatial information of the entire known transcriptome in just eight rounds of hybridization and four dyes (4^8^ = 65,536) (77, 78, 79). Realistically, this level of coverage is not achievable with the exponential increase in error rates per round of hybridization. MERFISH employs an error-detection barcoding scheme to account for a proportion of this error and when used in conjunction with bDNA probes to amplify the signal across ∼10,000 transcripts by 10.5-fold (79). Optical overcrowding of transcripts is also a limiting factor for such techniques. seqFISH+ was developed to circumvent this optical overcrowding by expanding the fluorophore palette from 4 to 5 colors to 60 “pseudocolors” using molecular barcoding, allowing analysis of 24,000 genes in four rounds with one round of error correction (80). MERFISH and seqFISH have provided insight into the spatial organization of the cell cycle, mouse hippocampus, and tissue development and homeostasis, as well as capturing nascent transcription active sites of genes (81, 82, 83, 84). Amplification is very powerful, though it only provides a global increase of intensity across targets, which cannot distinguish real RNA spots/signals from nonspecifically bound probes, which affects the resolution. To overcome this, experimentation with different split probes was conducted to achieve impressively punctate transcript spots, which can only fluoresce when two probes dock within immediate proximity on a highly specific and shared bridge sequence (85). An untargeted alternative to the aforementioned, fluorescent *in situ* sequencing (FISSEQ) used crosslinking and reverse transcription of RNA *in situ* to perform RNA-Seq with cyclic fluorescent probe ligations directly on the sample, which was measured *via* confocal microscopy (86). This method was demonstrated in a variety of sample types, such as primary fibroblasts, tissues, and whole embryos, and could be powerful in applications such as cellular phenotyping and gene regulation. The original FISSEQ publication uncovered that long noncoding RNAs preferentially localize in the nucleus. The premise of this method is powerful, but FISSEQ struggles to attain read counts comparable with standard single-cell RNA-Seq (scRNA-Seq), is difficult to perform in tissues, and is limited to short reads (86). A new variation of FISSEQ, known as In Situ Transcriptome Accessibility sequencing, has recently been developed for longer reads (87). To determine the precise subcellular localization of transcripts, it is recommended that organelle-specific dyes or immunofluorescence or organellar proteins are used as counterstains in these approaches (86). The versatility of FISH shows that it still has untapped potential in the transcriptomics world, and some of the newer methods have been recently reviewed (88).

#### Visualization Using Fluorescently Tagged Proteins

##### FPs

Genetically fused FPs are the next most prolific method of fluorescently labeling molecules, with the work that allowed scientists to harness FPs for research winning the Nobel Prize in chemistry in 2008 (89, 90, 91). Since the discovery and enhanced engineering of FPs, their use has provided immense biological insights into multiple processes, including demonstrating pH- and receptor-dependent endocytic viral entry during severe acute respiratory syndrome infection (92). This strategy involves fusing a reporter protein gene, usually an FP or a sequence that can be fluorescently labeled downstream, to a protein of interest using transfection. When the protein of interest is expressed, so is the fused reporter protein or sequence, which can then either be directly excited at the appropriate wavelength or labeled with a fluorophore (*e.g.*, a fluorescent antibody) (Fig. 1*B*). In contrast to strategies with affinity reagents, FPs allow for live-cell imaging, capturing temporal protein dynamics. An innovative and multicolored system called fluorescent ubiquitination-based cell cycle indicator utilizes fused FP monomers to two proteins, Cdt1 and geminin, that are specifically degraded in different parts of the cell cycle, at S/G2 and M/G1 phases, respectively. This strategy allows for cell cycle–dependent multicolored labeling of the nuclei (93). The strategy has allowed for deconvolution of cell cycle states and cellular processes that are otherwise difficult to distinguish. For example, it has been used to determine the relationship between the progression of double-stranded break repair and cell cycle status in living cells with the aim to help development and assessment of cancer therapies (94). However, sensitivity can be an issue, as it has been shown that only a third of the most abundant proteins in mammalian cells can be detected using the most widely used FP, GFP, although this can be mitigated *via* using more photostable or/and brighter tags (95). Furthermore, it has been shown that in certain cases, tagging endogenous proteins can interfere with specific properties of native molecules, including its subcellular localization. For instance, FPs have been found to erroneously locate at the endomembrane system of mammalian cells (96, 97). This localization artifact can be influenced by where the FP has been genetically encoded on the target protein (*e.g.*, on the N or C terminus). This effect was extensively examined in budding yeast (98). As well as this, protein fusion can also impair the normal expression, function, or degradation patterns of the native protein. Therefore, verification is required to ensure that endogenous localization and expression of the target molecule is unaffected by genetic fusion.

*Saccharomyces cerevisiae* has highly efficient homologous recombination processes compared with mammalian cells, making it relatively easy to generate FP-fused libraries, while generally preserving the normal expression patterns of the endogenous genes. Therefore, this species was used to conduct the first genome-wide library of a eukaryote for live-cell imaging using GFP tagging, achieving systematic localization of 75% of the yeast proteome to 22 distinct subcellular niches under normal culture conditions. This study provided novel localization information on 1630 proteins (99). Subsequent studies have used this yeast library under multiple conditions of environmental stress to uncover yeast protein localization dynamics as well as providing a quantitative dimension (100, 101, 102, 103, 104) (reviewed in Ref. (105)). Improved technology has led to further ease with creating genome-wide fluorescent fusion libraries. For example, the SWAp-Tag method, which allows efficient modification of a parental library and was employed for generating both an N-terminally–tagged and C-terminally–tagged yeast proteomes (106, 107, 108). Such extensive and numerous libraries enabled meta-analysis of protein localization dynamics in a quantitative manner with an unsupervised computational method (109). Such approaches have been able to differentiate perturbation-specific relocalization events from more generalized stress responses, concluding that protein subcellular localization provides an important layer of cellular regulation, independent from modulation of protein expression levels (17, 109). Because of the efforts mentioned previously, several databases containing imaging data on the spatial organization of the *S. cerevisiae* proteome are now publicly available (110, 111, 112, 113, 114, 115).

Similar efforts to systematically probe human protein subcellular localization using fluorescent reporter fusions have also been published but so far have only covered a small proportion of the proteome. For example, a collection of N-terminal and C-terminal GFP fusions to complementary DNA was generated to study protein localization in living human cells, resulting in localization assignment for 1600 human proteins (97). Similarly, an annotated reporter clone collection was built *via* exon tagging using retroviral particle–mediated delivery in 2006 (116). This collection has been used in combination with time-lapse fluorescence microscopy to track the abundance and localization dynamics of more than 1,000 endogenous proteins in living human cells under different conditions (116, 117, 118, 119). More recently, 1,311 proteins were fluorescently tagged using CRISPR-based fusion in multiple cell lines to achieve deep profiling of these proteins using 3D confocal microscopy, immunoprecipitation–mass spectrometry (MS), and next-generation sequencing (120).

The fluorescent tagging methods described previously center on protein labeling, but variations of these approaches have also allowed probing of RNA localization. Typically, this has been possible by encoding RNA hairpins into the gene of interest, which when transcribed can then be targeted by a corresponding RBP that is coexpressed and fused with FPs (121). The first and most used system of this kind is the MS2 system, which uses bacteriophage MS2 coat proteins (MCPs), which are RBPs, to target genetically inserted MS2 loops (122). Similar systems exploiting FPs have been added to the RNA localization repertoire, such as the P77 bacteriophage coat protein (PCP) system (123, 124, 125, 126, 127, 128). tdTomato-labeled PCP was used to successfully track individual mRNA molecules during translation at polysomes in different subcellular locations in dendrites (129). This study also utilized SunTag molecules, which provide protein scaffolds for multimerization of fluorescent tags to boost poor signal and to study translation in real time (129, 130, 130). Several other methods have been developed for studying translation in both fixed and live-cell applications, which are reviewed in detail (131). Single-molecule imaging of both translation and degradation in live cells can be achieved using the entertainingly named translating RNA imaging by coat protein knockoff (TRICK) and 3(three)′-RNA end accumulation during turnover (TREAT) methods, both of which use dual-color MCP and PCP reporter systems. TRICK can distinguish untranslated from translated transcripts by incorporating loops for MCP and PCP at different locations in the sequence of the mRNA of interest (132). During translation, the ribosome knocks off PCP in the coding region of the transcript leaving MCP behind (133). TREAT uses a similar concept, where PCP is used to label the 3′ end of the transcript, which is lost during degradation (134). Both TRICK and TREAT have been used independently in HeLa cells under arsenite stress to show reporter mRNAs retained in P-bodies are suspended, neither being translated nor degraded (134, 135, 136).

MS2-based and MS2-like systems tended to suffer from low signal-to-noise ratios. Constitutive fusion of coat proteins to FPs means that the fluorescence is independent of being bound to the sequence of interest. The signal-to-noise ratio can be significantly improved by including a nuclear localization sequence, so unbound protein is sequestered in the nucleus to improve the background of cytoplasmic transcripts (121, 137). Also, much like FP tagging, there is no clear rule as to where to genetically encode the RNA–stem loops within the endogenous transcript (138). There has been evidence that introduction of MS2-coated stem loops in yeast causes inhibition of mRNA decay, leading to RNA fragments that can continue to fluoresce and consequent aberrant localization measurements (139, 140). However, there is debate whether this evidence was an artifact of gene expression and/or the methods used to assess this degradation (141). To address these concerns, a modified coat-protein reporter system allowing for efficient RNA degradation was established in both yeast and mammalian cells (142).

##### RNA Aptamers

RNA aptamers have been used in both *in vitro* and *in vivo* imaging as affinity reagents and reporter tags, respectively (143, 144). They are short RNA oligonucleotides that can be conjugated to fluorescent dyes or designed to bind and induce the fluorescence of exogenous small molecules such as 3,5-difluoro-4-hydroxybenzylideneimidazolidinone (DFHBI), which is structurally related to the GFP chromophore (145) (Fig. 1*C*). DFHBI is structurally unstable, preventing its fluorescent activity until it is bound to the complementary active site of the fluorogenic RNA aptamer, bypassing the constitutive fluorescence that is caused by the persistent RBP–FP interaction in MS2-style systems.

The original DFHBI-binding RNA aptamer, Spinach, demonstrated excellent brightness with minimal background fluorescence and resistance to photobleaching. Typically, fluorogenic RNA aptamers are expressed fused to an RNA of interest for subcellular RNA imaging in live cells (143, 145). Guet *et al.* used spinach to show nuclear relocalization of *STL1* and CTT1 transcripts in *S. cerevisiae* upon osmotic stress (146). Conversely, cyanine-conjugated RNA aptamers have been used as affinity reagents for live-cell imaging of proteins including epidermal growth factor receptor, human retinoblastoma protein, and transferrin (144, 147, 148, 149).

In comparison to antibodies, aptamers have improved versatility with flexible modifications, less batch-to-batch variability, less steric hindrance, and are capable of labeling both nucleic acids and proteins (144). However, spinach, plus other RNA aptamers, have had issues with RNA degradation, intracellular folding, and thermal stability. Further aptamers, such as spinach2 and broccoli, have been designed to overcome these complications (150, 151). Additional fluorophores with corresponding fluorogenic aptamers have been designed to cover more of the visible and near-infrared spectra (152, 153, 154). Indeed, near-IR aptamers were the first to be adapted for live-cell super-resolution RNA imaging and have been used to detect subnuclear RNA structures in mammalian cells (154, 155). For further reviews of RNA aptamers, see Refs. (156, 157, 158).

#### Imaging Flow Cytometry

**Fig. 2:**
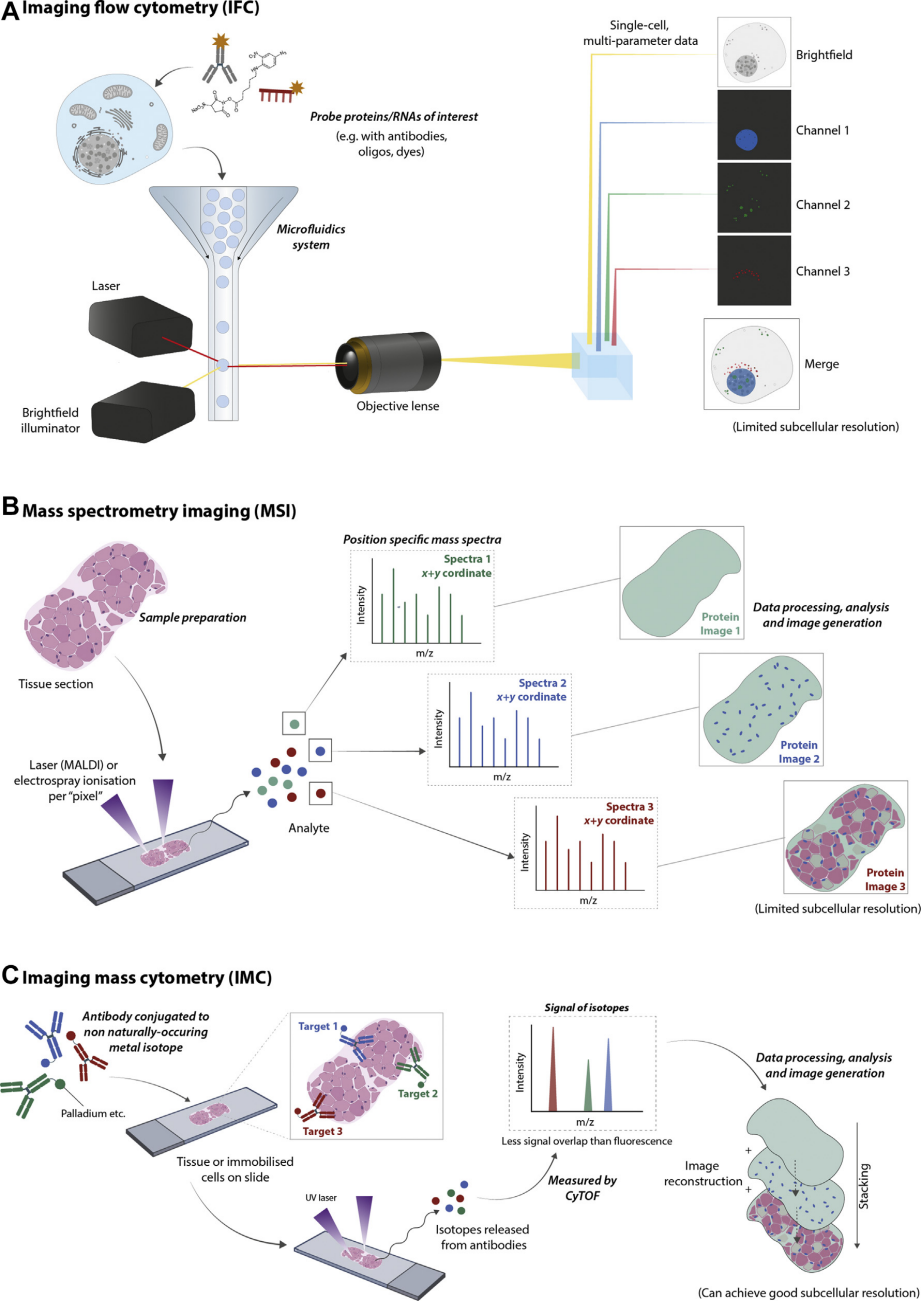
**Alternative imaging for subcellular proteomics and/or transcriptomics, which couple technologies in MS, microfluidics, and/or microdissection.***A*, instrumentation coupling flow cytometry and microscopy allows for multiplexing of several protein–RNA targets using fluorescent labels, gaining both spatial and single-cell information. *B*, microlaser ablation and ionization of molecules, such as peptides, lipids, or metabolites, directly from tissue or cell culture sample enables label-free acquisition of mass spectra across each “pixel” of sample. Very rich datasets but still have poor resolution because of current technical limitations. *C*, similar to MSI, microlaser ablation allows for acquisition of spectra per “pixel” of a sample. Though, this method has improved subcellular resolution and uses labeling of antibodies conjugated to non-naturally occurring metal isotopes to quantify ∼40 target proteins/RNAs of interest. The metal isotope signals have less signal overlap than fluorescent methods allowing improved multiplexing than traditional antibody probing. MSI, MS imaging.

Imaging flow cytometry (IFC) could be considered an alternative microscopy-based technique and can achieve up to 20 nm resolution (Fig. 2*A*) (159). IFC combines the multiparameter capabilities of flow cytometry and the morphological and subcellular spatial capabilities of microscopy (including dark field, light field, and fluorescence). However, in IFC, there tends to be a trade-off between throughput, sensitivity, and spatial resolution. To compensate for this, a technique to control the flow of cells in the microfluidics system was used to virtually “freeze” cells on the image sensor enabling longer exposure times in image acquisition (159). This improved signal-to-noise, throughput, sensitivity, and resolution. Whilst IFC cannot perform super high-resolution imaging and capture more intricate subcellular features, its application has been particularly useful for rare cell events and in diagnostic contexts (160). For example, it has been used as a diagnostic tool in acute leukemia to assess promyelocytic leukemia protein bodies and the cytoplasmic *versus* nuclear localization of a characteristic antigen (161). Another major consideration is that the approach requires cells to be in suspension, and dissociation of adherent cells or tissues may cause aberrant localization of molecules. Whilst performed less frequently than protein analysis, RNA transcripts can be visualized using IFC (162, 163).

### Non-microscopy-Based Imaging Methods

Imaging techniques that do not rely on microscopy are also available to map subcellular localization. These typically consist of hybridizing flow cytometry and/or MS to imaging. Whilst exciting, their use is still limited. Therefore, we only briefly provide an overview but direct to relevant sources of further reading.

#### Imaging Mass Cytometry

Imaging mass cytometry (IMC) uses a similar instrumental setup to mass cytometry, which hybridizes flow cytometry and MS using a cytometry by time of flight (cyTOF). This technology does not suffer from the same degree of signal overlap compared with fluorescent tagging systems (164, 165). The MS element allows discrimination between targets at an isotopic scale. This is achieved by coupling probes, commonly antibodies, to discrete heavy-metal isotope tags (164, 166). Currently, this tagging system allows around 40 targets of interest to be measured per single cell. In traditional mass cytometry, cells are passed through a microfluidics-style droplet system and through argon plasma at a high temperature when entering the instrument where covalent bonds within molecules are broken, releasing free atomic-level ions. The ions enter a quadrupole where the heavy-metal isotope tags are selected. These tags go on to be separated by mass-to-charge in the cyTOF component of the instrument (164, 166). This is a destructive process, so cells cannot be sorted *via* this technique, unlike flow cytometry sorting methods (*e.g.,* fluorescence-activated cell sorting), and spatial information is lost. IMC overcame this loss of spatial information by coupling laser ablation of tissue slide or cell culture a pixel at a time into a cyTOF (Fig. 2*C*). In the first publication of this method, the ability to untangle the heterogeneity of breast cancer samples was demonstrated (166). A similar study recently claimed to achieve subcellular resolution using IMC for 37 proteins in 483 breast cancer tumors to assess the phenogenomic correlation with protein expression (167). Breast cancer samples were also used to simultaneously image 16 proteins and three mRNA targets using a combination of antibodies and oligonucleotide probes, respectively (168). A variant of IMC was developed, which employed an ion beam to liberate metal ion reporters, known as multiplexed ion beam imaging, which increased speed, sensitivity, and resolution, and has been reported to give “super-resolution” images of 5 to 30 nm (169, 170). Currently, IMC-like strategies have been used successfully for cellular phenotyping of lesions in multiple sclerosis and lymphoid organs, primarily at a tissue level rather than subcellular level (171, 172). Yet their capabilities for providing such resolution are coming into fruition.

#### MS Imaging

IMC may be confused with MS imaging (MSI), though MSI differs in instrumentation and does not require heavy isotope–derivatized antibody labeling. As with IMC, laser ablation is used to ionize individual “pixels” of a sample, with each pixel having a corresponding label-free spectrum, which allows deeper coverage of molecules than IMC (Fig. 2*B*). However, the technique suffers from poor sensitivity and resolution (commercial instruments ranging from 5 to 20 μm), so is predominantly only useful for macroscopic imaging where subcellular resolution is not in the scope of the experiment (173, 174, 175), although hybrid MS setups have allowed this technology to improve its resolution. For example, researchers mixed and matched ion sources, such as atmospheric pressure and laser-induced postionization (MALDI-2) sources, coupled to orbitrap analyzers to achieve 1.4 and <1 μm resolution, respectively (174, 176). Because of MS vulnerability to contaminants, a lot of sample preparation methods, such as fixatives, are incompatible with this method and often flash-freezing is preferential, but further MS-friendly methods are under investigation (173). Currently, MSI still suffers from shortfalls in achieving subcellular resolution; so there is limited discussion in this review, and more comprehensive details of MSI can be found (173, 174, 175). Arguably, MSI has yet to be fully integrated within subcellular -omics workflows because of limited resolution, but advances in the technologies associated with the approach promise to improve the general utility of the MSI. Currently, MSI has been considered for tissue-level intraoperative imaging, particularly on difficult to image and difficult to measure biomarkers in pancreatic adenoma (177, 178). What makes this approach particularly exciting is that it can be applied to any molecules that can be ionized, which include proteins, metabolites, or lipids.

## Sequencing-Based Methods in Spatial Transcriptomics and Proteomics

In the postgenomic era, advances in MS-based and RNA-Seq-based technology have allowed researchers to simultaneously quantify thousands of proteins and RNA species in whole cells and tissues. Along with the concurrent advancement of computational tools, powerful spatial -omics workflows can analyze the structure and molecular composition of specific or several subcellular compartments in one experiment. The methods in this section provide spatially enriched samples of proteins or RNA on a subcellular level that are measured downstream using MS or RNA-Seq. Generally, these methods eliminate *in situ* spatial information during sample preparation and capture “bulk” information of all cells within a given sample. Therefore, achieving single-cell information using the following methods is still challenging, particularly in proteomics because of the inability to amplify proteins (179, 180, 181, 182, 183, 184, 185). Details on the type of MS and RNA-Seq approaches that can be coupled with the methods in this section are reviewed (186, 187, 188).

### Biochemical Separation

Established techniques that enrich or isolate cellular structures by their physicochemical properties have been in use for decades. Typically, subcellular distribution of molecules was assessed using target-specific enzymatic assays (189), whereas modern techniques employ robust quantitative sequencing using RNA-Seq and MS (186, 187, 188).

#### Basic Centrifugation-Based and Detergent-Based Fractionation

Centrifugation is one of the simplest methods to separate organelles based on their size, density, and shape. Organellar preparations using centrifugation date back to the late 1800s, initially to isolate nuclei (190, 191, 192). Today, there are two generalized categories of centrifugal organellar fractionation, sedimentation, and equilibrium density centrifugation. These result in either an enriched pellet at the base of the tube or at the organelle's equivalent density within a sucrose (or equivalent) gradient, respectively. When coupled with current sequencing technologies, these enrichment strategies are powerful for exploring subcellular composition.

**Fig. 3:**
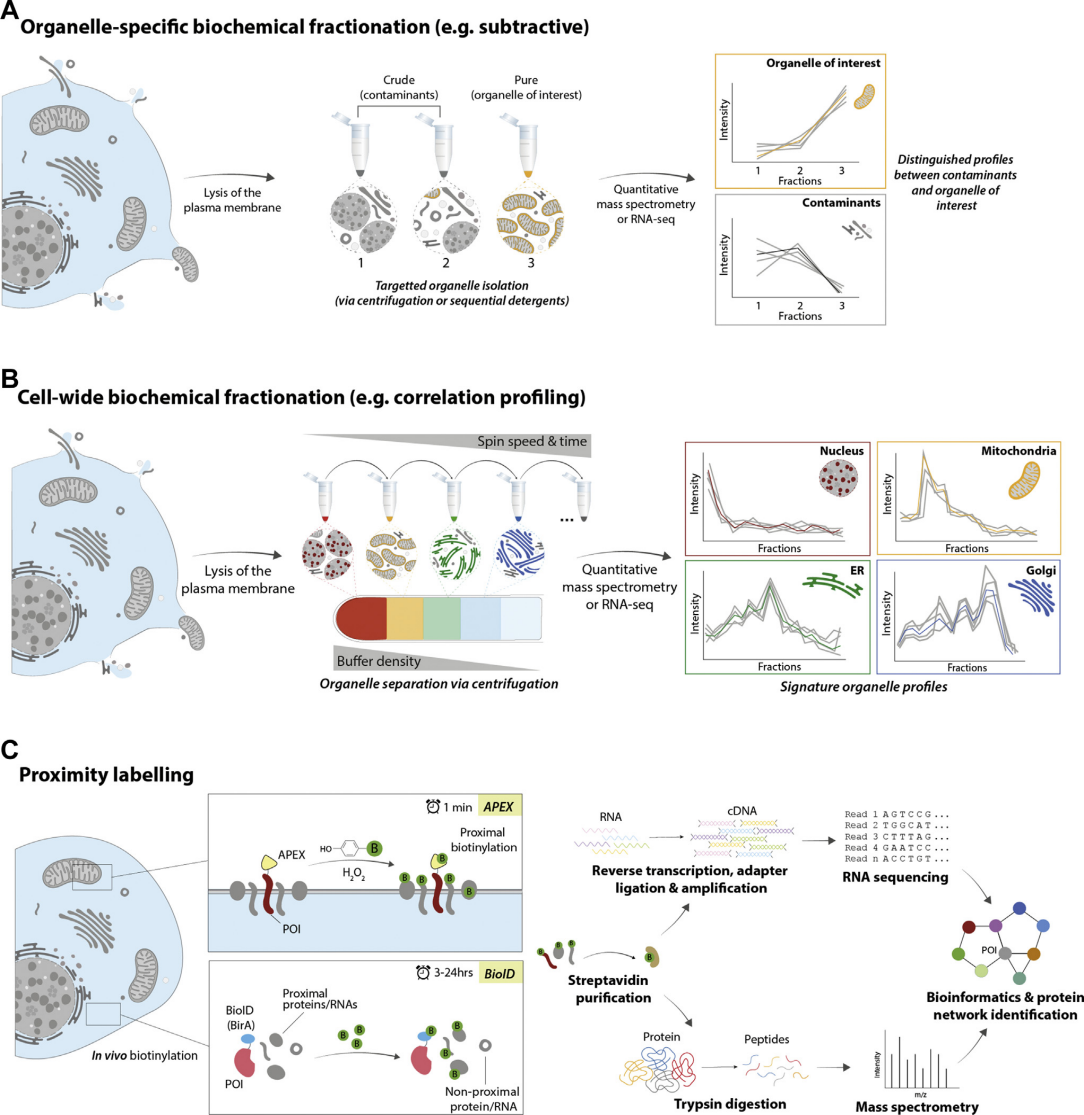
**Sequencing-based approaches to subcellular proteomics and transcriptomics.** The approaches consist of biochemical organellar separation (*A* and *B*) or biotinylation of proximal molecules to a bait protein (*C*). *A*, quantifying proteins/RNAs in a targeted organelle-enrichment preparation (*via* centrifugation or detergents) against crude contaminant samples can infer resident proteins/RNAs of the organelle of interest. Quantification of enriched samples can be performed using MS or RNA-Seq. *B*, more extensive sequential centrifugation or detergent strategies can determine cell-wide residence of proteins/RNAs. The quantitative profiles of proteins/RNAs across the fractions aid identification of their localization by using organellar markers and machine learning techniques. *C*, a bait protein of interest (*e.g.*, associated with a particular subcellular localization) is fused to an enzyme that catalyzes the biotinylation of proximal proteins/RNAs in the cell once the substrate (*e.g.*, biotin) is added to the cells *in vivo*. The biotinylated molecules can be purified and analyzed using either MS or RNA-Seq.

Early spatial proteomics studies focused on purification of a singular organelle of interest, giving insights into the molecular composition of many cellular compartments, such as the nucleolus, nucleus, nuclear pore, and mitochondria, across many cell/tissue types and models (193, 194, 195, 196). However, purifying subcellular compartments is challenging because of cofractionation with other components of the cell, because of organelles having overlapping biochemical and biophysical properties, and their constant interaction with one another. “Subtractive” or “differential” approaches account for this “contamination” or interactions. These methods involve quantitative comparisons of technically equivalent non-enriched fractions against organelle-enriched fractions (Fig. 3*A*). Proteins only detected or highly enriched in the organelle-enriched fractions are assigned to that organelle of interest. This strategy has provided valuable information on the subcellular proteomes of the human spliceosome (197, 198), rodent liver nuclear envelope (199), rat lung endothelial cell PM, and caveolae (200, 201), plus multiple subcellular niches in *S. cerevisiae* and other yeasts using diverse enrichment approaches (202, 203, 204, 205, 206, 207, 208, 209). However, despite accounting for contaminants, it is still difficult to confidently identify organellar proteins, as the composition of any cofractionating organelle will be erroneously assigned to the organelle of interest. In addition, this technique is not always appropriate for multilocalized molecules or dynamic studies (210). Coupling of subtractive proteomics with machine learning has improved classification of organellar proteomes (211, 212), which somewhat mitigated this issue by providing more robust statistical comparison between enriched and non-enriched fractions. These strategies have been used to establish biological functions and confident inventories of organellar proteomes, such as the mitochondria, peroxisome, and lysosome (213, 214, 215, 216, 217, 218). Such studies can be particularly useful for poorly characterized species, such as eukaryotic parasites, with the intention to aid biological understanding and pharmacological developments. For example, this strategy was used to assess the proteins involved in the mitochondrial “importome” of *Trypanosoma brucei* by coupling with RNA interference of a key translocase (219).

Organelles can also be enriched using different detergent-containing buffers with increasing solubilization capacity to sequentially extract molecules from distinct parts of the cell (220). For instance, the use of digitonin to permeabilize the PM or NP-40 to release contents of double-membrane organelles. The most popular workflow in proteomics achieves subcellular separation of the cytosol, nucleus, cytoskeleton, and membranous compartments (such as those found in the secretory pathway) (221). Modified protocols can further distinguish between DNA-associated and soluble nuclear proteins or insoluble proteins in the cytosolic, nuclear, and membrane-bound components (222). This approach was implemented in a phosphoproteomics study to resolve three crude subcellular compartments with a very limited amount of starting material (223). Notably, detergent enrichment workflows have the advantage of preserving the cytoskeletal network, which is prone to fragmentation in centrifugal fractionation (221). Differential detergent extraction is primarily reserved for proteomic studies. However, it has been used for studying polysomal RNA and in a two-step detergent protocol to investigate cotranslational trafficking of mRNA from cytosolic polysomes to ER-bound polysomes (224, 225).

The development of equivalent biochemical fractionation methods to determine subcellular RNA localization is, in comparison, limited. Several studies use basic cell fractionation *via* centrifugation and detergent lysis followed by RNA-Seq to infer transcript subcellular enrichment (226, 227, 228). A sequential detergent strategy was employed to map spatial dynamics of RNA between the cytosol, nucleoplasm, and chromatin in inflammatory-stimulated macrophages by assessing the relative enrichment of transcripts in the different fractions to gain insights into proinflammatory gene regulation (226). Similar protocols have been applied to obtain a static distribution of transcripts within these compartments in human embryonic kidney 293 cells (227, 228). Generally, subcellular enrichment can be assessed *via* Western blot by using antibodies against corresponding organellar proteins (229). However, this assumes that RNA species cofractionate with their associated cellular compartment in the same way as proteins, although, proteins and RNA behave differently under centrifugation (217, 230, 231). This may be due to the variability of sedimentation rates and aggregation of RNA molecules, which is dependent on the concentration of salts they are present in. This behavior of RNA is believed to have caused artifacts in early attempts at RNA purification and fractionation, leading to controversy over estimates of sedimentation coefficients for RNA molecules (232, 233, 234). The cause of these artifacts may be explained by the size difference between proteins and mRNA, where the transcripts are orders of magnitude larger than proteins (235). Cytosolic RNA may have similar buoyancy and sedimentation as some subcellular niches, particularly if they aggregate, leading to erroneous assignments of RNA species to various subcellular compartments. These protocols may be improved by including a similar quality control step involving quantitative PCR for transcripts with known localizations.

Furthermore, the methods focus on enriching only a few compartments, which is also a problem shared with the subtractive proteomics methods mentioned previously (226, 227, 228). The limited cellular compartment coverage for assessing RNA localization is partially addressed by the CeFra-Seq method, which covered five fractions: nuclear, cytosolic, endomembrane, insoluble, and extracellular (236). In this workflow, differential and density centrifugation were coupled with targeted detergent permeabilization (230, 236). The intention of this method is to enable measurement of global changes in RNA trafficking upon genetic or environmental stimuli. While these methods have provided important contributions to the understanding of components and functions of cellular architecture, they were too crude to resolve multiple organelles within the same experiment, particularly those compartments that are biophysically similar or highly interconnected, such as the secretory pathway.

#### Protein–RNA Correlation Profiling

To address the limitations of more reductive centrifugation methods, correlation profiling was developed based on the principles of Christian de Duve, where localization of proteins can be determined without organellar purification (189). Protein correlation profiling involves organellar enrichment using a density gradient alongside quantitative MS to measure abundance of peptides across the gradient. Localization of proteins is then inferred by comparing their gradient distribution patterns to those of known organelle marker proteins, usually performed using computational machine learning strategies, such as support vector machine classification (Fig. 3*B*) (237, 238, 239, 240). Protein correlation profiling was originally applied to single compartments of interest, such as the centrosome, peroxisome, lipid droplet, and proteasome in various model organisms (241, 242, 243, 244). The technique was expanded for global organelle analyses in multiple mouse tissues (245, 246). Another density gradient centrifugation technique, localization of organelle proteins by isotope tagging (LOPIT), employed isotope-coded affinity tagging to multiplex the gradient fractions and map the global subcellular proteome of the *Arabidopsis thaliana* root-derived callus material (247, 248). Since then, LOPIT has evolved alongside isobaric tagging technologies, allowing the study of the subcellular proteomes of diverse model systems, including human cell lines, chicken lymphocytes, and *Drosophila melanogaster* embryos (249, 250, 251, 252). A further evolution of this protocol, hyperplexed LOPIT, used a more complex density gradient to study pluripotent E14TG2a mouse embryonic stem cells and U-2 OS human bone osteosarcoma cells, which demonstrated highest subcellular resolution than any other MS-based spatial proteomics method available to date (40, 253). Hyperplexed LOPIT has also been employed to comprehensively map the subcellular organization of *S. cerevisiae*, cyanobacterium (*Synechocystis*), and *Toxoplasma gondii* (254, 255, 256). The method has also been coupled to free-flow electrophoresis (FFE) (see Imaging Flow Cytometry "Electrophoresis-Based Methods" section) to analyze the protein composition of Golgi subcompartments in *A. thaliana* cell-suspension cultures (257). These comprehensive datasets are designed to provide holistic catalogs of system-wide proteomes to provide biological insight of organellar components as well as the option to compare between systems and perturbations.

A similar complex density gradient was used to perform cell-wide temporal analysis of subcellular composition during human cytomegalovirus infection, capturing protein dynamics, providing unprecedented understanding of the organellar architecture of host cells during infection (237). Differential centrifugation and/or detergent strategies can also be coupled to this correlation profiling approach, which the following methods utilize: dynamic organellar maps, LOPIT-differential ultracentifugation, and SubCellBarCode (239, 250, 258). These methods vary in separation, labeling, and analysis protocols that range in resolving power and come with their own advantages or limitations, depending on the study design and biological questions being addressed, offering flexible and customizable options for researchers. These methods can achieve high coverage, often >8000 proteins, and some of these methods have achieved suborganellar resolution, such as resolving the ER–Golgi intermediate compartment, ribosomal subunits, chromatin, and subnuclear compartments (239, 253, 258). Dynamic organellar map has been used to further investigate protein trafficking after perturbation with compounds that enhance antigen import *via* lysosomal trapping (259).

The big challenge of these correlation profiling experiments is the data analysis of their complex, multidimensional datasets. Answering apparently trivial questions can become challenging, specifically identifying protein translocation events and proteins localized in multiple cellular compartments. However, novel computational models, such as TRANSPIRE, BANDLE, and MR scoring, have ventured to address questions on protein dynamics (260, 261, 239). T-augmented Gaussian mixture model approaches have been developed to tackle questions on multiply localized proteins (262, 263 ). A recent evaluation of some of these approaches can be found (264).

In transcriptomics, sucrose gradients are frequently used to assess mRNA association with polysomes, which can be separated into cytosolic- and ER–polysome-bound transcripts (225, 265, 266). It is thus reasonable to suggest that correlation profiling methods could be adapted for spatial transcriptomics assigning RNA subcellular localization based on distribution profile comparisons to curated “RNA markers” of known localization. Current methods are typically protein centric or are based on separating RNA along with their protein interactors, for example, within stress granules or ribosomes (267, 268, 269). New approaches, such as ATLAS-Seq, have been able to cosediment different RNAs using density gradients coupled to RNA-Seq and then use hierarchical clustering to infer subcellular localization of transcripts (231). When mapping the transcriptome of mouse liver, the authors found that transcripts that cosediment tended to encode proteins that coassociate, including proteasomal subunit mRNAs. In addition, alternatively spliced transcripts typically showed differential sedimentation patterns. However, transcripts with similar correlation profiles did not consistently colocalize when interrogated with an orthogonal method such as single-molecule inexpensive FISH, indicating need for further improvement of such approaches (231). Thus, there is a current gap in technology and a requirement to develop a spatial transcriptomics technique that truly complements protein correlation profiling.

The key benefit of protein correlation profiling–based methods is the ability to interrogate all cellular compartments at once and therefore be able to address dynamic and complex biological problems, which the majority of the other techniques discussed in this review struggle to do on the same scale. The primary drawback of all protein correlation profiling methods is that they capture “average localization” of proteins within a given sample. Therefore, data from samples that are likely to contain a heterogeneous population of cells may be more complicated to interpret. Examples of such samples include tumor cell lines in different cell cycle stages or cell types within a tissue. Techniques to resolve this, such as cell cycle synchronization or microdissection, reduce input material, which could complicate downstream sample preparation. Another consideration is that mechanical or chemical bursting of the outer membranes of cells is required for protein correlation profiling, which may cause artifacts, such as lost interactions or leakage from organelles and organellar membranes.

#### Electrophoresis-Based Methods

Another biophysical property that can be exploited for organellar fractionation is charge states. FFE relies on the same principles of electrophoresis, where particles in a biological sample are separated *via* their surface charge densities. It is a versatile technique that can separate a variety of charged analytes including low molecular mass organic compounds, proteins, peptides, macromolecular complexes, organelles, and whole cells under native or denaturing conditions in aqueous separation buffers. Notably, FFE-mediated cell fractionation experiments are characterized by fast separation and high sample recovery rates. Furthermore, FFE can be paired with various tools, such as specific antibodies, lectins, chemical ligands, and proteases or other enzymes to optimize organelle separation, by introducing subtle changes in the surface charges of certain compartments with minimal disruption to their functional integrity (221, 270, 271, 272, 273). FFE has been used in combination with centrifugal separation and MS analysis to resolve subpopulations of organellar networks that are otherwise difficult to capture, including the PM, components of the ER network, endosomes, lysosomes, phagosomes, peroxisomes, mitochondria, and plant tonoplasts (vacuole membranes) (221, 270, 271, 272, 273, 274, 275, 276, 277). De Michele *et al.* were able to assign peripheral membrane proteins that are normally lost in traditional PM enrichment to the *Arabadopsis* PM, including the entire exocyst complex. This method has been used to separate DNA in a size-dependent manner but has yet to be used to study RNAs, particularly in a subcellular context.

Flow field-flow fractionation (FIFFF) is similar to FFE but instead uses a “cross-flow” system that drives separation in a shape- and size-dependent manner, providing distinct elution patterns for different sample constituents (271, 278). FIFFF with MS has been used to analyze subcellular structures, such as the mitochondria, extracellular vesicles (EVs), and lipoprotein particles (278, 279, 280). The technique was used to separate and define a new subpopulation of small EVs termed exomeres, which are selectively enriched in glycolytic and mammalian target of rapamycin signaling proteins compared with larger EVs (281). This method has also been used in a cell-wide context demonstrating simultaneous separation of multiple human subcellular compartments, albeit with lower resolution than centrifugation strategies (282).

Much like standard scRNA-Seq, microfluidics-based electrophoresis has also been exploited for subcellular scRNA-Seq. Single-cell integrated nucRNA and cytRNA sequencing (SINC-Seq) captured single cells using a hydrodynamic trap, followed by selective electrolysis of the PM to attain intact nuclei (283, 284). The cytoplasmic RNA was then separated based on ionic mobility *via* electric field activation and used to construct individual RNA-Seq libraries. SINC-Seq in K652 cells showed that over pseudotime, the differentially expressed genes in cytRNA *versus* nucRNA showed less correlation between the two compartments when histone acetylation was perturbed using sodium butyrate. Impressively, this method resulted in only a 5.3% drop in reads when compared with standard scRNA-Seq. In NanoSINC-Seq, the microfluidic fractionation is coupled to Nanopore complementary DNA sequencing to compare isoform diversity between the cytoplasm and nucleus (285). There is potential for the systems harnessed in FFE and FIFFF to be modified for single-cell sequencing of other organelles (284).

### Proximity Labeling–Based Methods

Proximity labeling was originally developed for capturing the interactomes of specific proteins *in vivo* followed by downstream purification. Therefore, mapping interactomes of multiple bait proteins can essentially capture the “local” spatial proteome or transcriptome of each bait (286, 287, 288). Proximity labeling workflows consist of fusing a bait protein of interest to an enzyme, typically a biotin ligase or a peroxidase, which covalently labels proteins and RNA in the immediate vicinity of the bait with a small and exogenous substrate, typically biotin (Fig. 3*C*). Because of the short half-life of the substrate, only molecules within a few tens to hundreds of nanometers of the bait are labeled. Therefore, there is no reliance on direct physical interactions (286).

Proximity labeling overcomes shortcomings of traditional affinity purification protocols where interactions can be disrupted during sample preparation, with the caveat of contamination with promiscuous cellular components, such as diffusing components of the cytosol and background biotinylation. However, these promiscuous molecules can be mostly accounted for with the appropriate controls and by referring to a contaminant repository for affinity purification experiments, known as the CRAPome (http://crapome.org) (289, 290). Other considerations of proximity labeling include the variable elution of enriched molecules from affinity matrices, changes in expression, localization or function of the fused bait protein, and that amino acid residues targeted for biotinylation by the fused enzyme must be present on the surface of the proximal proteins. Therefore, proteins or RNA lacking these residues on the surface of their structure would be missed, and it has been shown that proximity labeling favors intrinsically disordered regions where these residues are more likely to be exposed (291). In addition, compartments with highly dynamic and soluble molecules, such as the cytosol or nucleoplasm, are difficult to target using this strategy, because these subcellular niches do not offer a small, defined, and membrane-enclosed space to which the bait protein can be specifically targeted, resulting in high rates of nonspecific biotinylation. Multibait strategies for assessing subcellular localization across several organelles have started gaining popularity. However, there is no guarantee that the fused enzyme will have comparable activity in these different subcellular locations. This has implications on the quantitation of the data, where true proportions of the proteins or RNA across these compartments cannot be deduced, and rather, a qualitative list of species in those locations is produced. In addition, this method is limited to biological systems that can be genetically engineered. Despite this, proximity labeling excels at capturing membrane-bound organelles and proteins associated with insoluble cellular structures, such as various cytoskeletal components, which are challenging to isolate and reliably analyze with alternative methods (292). Here, we cover the key enzymes used and how they are applied to both spatial transcriptomic and proteomic studies. The strategy has been invaluable for uncovering biological networks, albeit in a spatially restricted manner. Moreover, powerful cell-wide proximity tagging studies have recently started to emerge, as well as transcript-capturing approaches, indicating what the future holds for this approach (287).

#### BioID-Based Methods

BioID is a proximity labeling method that uses biotin ligases. Originally, wildtype BirA enzyme from *Escherichia coli* was used, which catalyzes biotinylation in the presence of ATP on molecules containing a biotin acceptor peptide (BAP) sequence (293). This restricted targeting to proteins with BAP regions and exposed lysine residues. BioID overcame this limitation by engineering BirA to promiscuously biotinylate proteins (294). This allowed for improved labeling efficiency, selectivity, faster incubation times, and higher signal-to-noise ratios. The newer generation of the BirA enzymes, such as TurboID or MiniTurbo, can achieve labeling within minutes rather than hours, enabling study of rapid and dynamic cellular processes (295, 296). These optimized enzymes have been recently reviewed (286).

BioID has been successfully applied to a range of interactions on a complex, organellar, and dynamic level in a variety of cell and tissue types, as well as entire organisms. In mammalian cells, BioID-based labeling strategies have provided insights into macromolecular complexes or subcellular niches, including the nuclear lamina, nuclear pore complex, nucleosome complexes, mediator transcription regulation complex, ER–peroxisome contacts, and focal adhesions (289, 294, 297, 298, 299, 300, 301). In addition, BioID has been used in applications beyond immortalized mammalian cell lines. For example, the approach was used to study three unique subcellular niches in *Trypanosoma brucei*, the basal body, flagellum, and bilobe, plus entire organisms, specifically flies and worms (295, 302, 303, 304), revealing novel organellar components of these organisms that are otherwise difficult to capture. Multibait studies were used to identify novel proteins at the centrosome–cilium interface and involved in ciliogenesis, to unpick the phosphorylation regulation in the Hippo signaling pathway, and the interactome of P-bodies and stress granules during normal and stressed conditions, using 58, 19, and 139 baits, respectively (305, 306, 307). Impressively, examples of cell-wide BioID experiments have recently emerged that have captured 26,527 and 35,902 interactions located within 21 and 32 distinct cellular features, respectively, with the former identifying a further 9,390 interactions when coupled with affinity-purification MS (287, 288). These studies demonstrate suborganellar resolution that is difficult to achieve *via* correlation profiling.

Despite working well in proteomics, BioID has not yet been adapted to directly label RNA for spatial transcriptomics studies. Studies that do involve targeting BirA enzymes to RNA are typically focused on determining the protein interactors of specific transcripts, rather than biotinylation of RNA in certain organelles (308, 309, 310). However, pairing BioID with ribosome profiling enabled identification of translated transcripts in a specific subcellular context. In this method, the biotin ligase was expressed as a fusion protein to localize it to the subcellular niche of interest, and ribosomes were expressed with a protease-cleavable BAP (AviTag). With addition of biotin, only ribosomes in the vicinity of the ligase were biotinylated, after which they were isolated, and the translating RNA was sequenced. Proximity-specific ribosome profiling was used to profile the translatome of the ER and mitochondria in yeast and human cells (311, 312, 313). In addition, BioID was adapted for *in vivo* purposes to study the inhibitory neuronal network and synapse formation in live mice identifying both known and novel proteins involved in the hyperpolarization process with some linked to neurodegenerative diseases (314, 315).

#### APEX-Based Methods

APEX is also a proximity labeling technique that allows for the mapping of the proteome and transcriptome with spatial and temporal resolution. Instead of the BirA derivatives used for BioID, APEX uses a modified soybean-derived ascorbate peroxidase. The enzyme catalyzes oxidation of a supplied biotin derivative (usually biotin–phenol) and generates spatially confined and short-lived biotin–phenoxyl radicals that react with electron-rich side chains of amino acids (such as Tyr, Trp, His, and Cys), resulting in covalent biotin labeling of proteins and RNA in the near vicinity. The primary advantage of APEX over BioID is that the tagging reaction is faster, allowing for dynamic experiments capturing discrete time points. Detailed comparisons of APEX and BioID, including their novel variants, can be found in recent reviews (286, 316).

The APEX-based system coupled to quantitative proteomics has been instrumental in exploring various subcellular compartments and networks. For example, its use in human cells allowed analysis of various mitochondrial subcompartments, the ER membrane, and the endocytic system, as well as investigating interaction of the interactions of bioactive peptides (or “microproteins”) (317, 318, 319, 320, 321, 322, 323, 324, 325). The advantages of APEX over BioID were epitomized in time-resolved studies. For example, APEX has been used to capture the transient interactome of G protein–coupled receptor signaling identifying novel regulators of the associated delta opioid receptor and β2 adrenoceptor (326, 327). Another temporal study using APEX showed unique and aberrant stress granule dynamics in cells of amyloid lateral sclerosis, helping identify novel disease-relevant protein candidates (328). An improved variant, APEX2, has allowed increased catalytic activity and labeling sensitivity compared with APEX. In contrast to the original monomeric APEX protein, APEX2 has the capacity of forming dimeric complexes, which has been shown to improve the activity as well as stability of ascorbate peroxidase enzymes (319).

APEX technology has been recently adapted for spatial transcriptomics. There are two main applications of APEX for determining RNA subcellular localization: proximity labeling of protein crosslinked to RNA and proximity labeling of the RNA nucleosides directly. The first method published was APEX-RNA-co-immunoprecipitation (RIP), which combined APEX-dependent *in situ* protein biotinylation with formaldehyde crosslinking of the labeled proteins to RNA in specific compartments or organelle interfaces. In a proof-of-concept study, APEX-RIP mapped transcripts associated with the nucleus, mitochondrial matrix, ER membrane, and cytosol in human cells (329). A related method, proximity-crosslinking and immunoprecipitation, achieved simultaneous profiling of both free and RNA-bound proteins at specific subcellular locations by combining APEX2-dependent protein tagging with UV-mediated protein–RNA crosslinking (330). APEX-RIP has the benefit of not requiring RNA labeling, whereas proximity-crosslinking and immunoprecipitation uses 4-thiouridine labeling of RNA to enhance crosslinking efficiency (330). However, APEX-RIP suffers from poor specificity in membrane-less regions of the cell because of the use of formaldehyde crosslinking (329). These problems were addressed by an alternative implementation of APEX for RNA localization called APEX-Seq (3, 331). This method took advantage of the discovery that the APEX2 enzyme directly labels nearby RNA as well as proteins. The biotinylated RNA could then be affinity purified with streptavidin beads and sequenced. The APEX-Seq approach was used to generate an atlas of human RNA localization covering nine different subcellular niches and to probe the spatial organization of transcripts associated with translation initiation complexes as well as repressive RNA granules (18, 331). This study unveiled the dynamic, varied, and stress type–dependent nature of stress granules. It is important to remember that APEX-Seq uses the same biotin–phenol substrate as standard protein proximity labeling, and subsequently, proteins are also biotinylated. Recently, it has been shown that using different biotin substrates, such as biotin–aniline, improved labeling efficiency of nucleic acids *versus* proteins (332). It is worth noting that biotin–phenol is toxic to cells at higher concentrations, which can limit its use in certain model systems, such as in tissue or whole organisms. BioID may be more appropriate in these experimental scenarios. Despite this, APEX-based labeling was adapted for application to *in vivo* model systems to map various subcellular compartments, such as nucleus-associated, mitochondrial matrix–associated, and Golgi apparatus–associated proximity networks, in live yeast, *Caenorhabditis elegans*, and *Drosophila* (333, 334, 335, 336, 337, 338).

## Future Prospects

This review demonstrates that there are several options available to researchers to address biological questions concerning the subcellular localization and trafficking of proteins and transcripts. However, the technical challenges can still be vast and differ between transcriptomics and proteomics, as well as the biological system and question in hand, which is the intrinsic reason why there is lack of a one-size-fits-all approach. Although there have been attempted hybrid methods where multiple probing across molecular species is possible, such as in IMC, these still often lack the same coverage or resolution as sequencing or microscopy (168, 173). Here, we briefly discuss the methods that attempt to address fundamental limitations hindering the field and what the future may hold for subcellular -omics.

MS-based, RNA-Seq-based, and imaging-based methods are continuing to make great advances in improving coverage and resolution. Instrumentation advances have allowed researchers to push the boundaries in subcellular resolution and coverage (187, 188, 339). In addition, complex multiplexing strategies, such as DNA-PAINT, and super-resolution imaging are becoming standard practice in more research laboratories (53, 54, 56, 78, 80). When combined, these techniques are not limited to studying localization in membrane-bound organelles but can be extended to imaging biomolecular condensates (340). Automation and artificial intelligence analyses are enabling deconvolution of vast quantities of imaging data to determine the extent of single-cell subcellular heterogeneity (10, 40, 341), plus detection of translocations and multilocalized molecules (260, 261, 263). Because of the impressive advances in methodologies and data analytics, scRNA-Seq is now relatively straightforward (181, 342, 341). However, gaining subcellular scRNA-Seq information is more challenging, with poor spatial resolution or poor read coverage, as seen from SINC-Seq and FISSEQ, respectively (284, 343). Single-cell proteomics using MS is similarly in its infancy, hindered by the lack of a PCR-like amplification available for proteins. Despite emerging strategies for single-cell proteomics and sensitivity of MS instrumentation, there is still a long way to go to extract subcellular information on a single-cell level (183, 344, 345). In addition, many of these methods are enrichment based and do not provide absolute quantitation, rather relative quantitation.

In addition to differential expression and subcellular distribution of RNA and protein, there are other dimensions of molecular biology that can influence physiological processes, subcellular localization, and states of cells, such as molecular structure, stability, turnover, interactions, and modification (*e.g.*, post-translational modifications [PTMs] and splicing). Current approaches often do not capture this information, which is vital to understanding control of localization and molecular roles of proteins and RNAs. Capturing this information in addition to spatial data may deconvolute some of the ambiguity found between datasets as it is still unclear how these molecular characteristics, such as PTMs, influence protein and RNA distribution. This is becoming more achievable with improved MS technologies and protocols. For example, recent improvements in cross-linking MS technology allows inference of interactions and protein structures/folding, *via* forming identifiable chemical bridges between residues (346, 347, 348, 349), and ion-mobility technology is improving throughput of “native, in-tact” proteins as well as improved identification of multiply phosphorylated peptides (350, 351, 352). Enrichment protocols and commercial kits are also aiding PTM analysis *via* MS with reduced starting material ( 353, 354). Nanopore sequencing is providing more straightforward and accurate RNA modification data, alongside other methods for RNA modification analysis, which include variants of next-generation sequencing and LC–MS/MS analysis (187, 355, 356). In addition, straightforward and simultaneous enrichment of proteins, RNA, and RBPs with crosslinking and phase separation, with reduced starting material compared with conventional methods, such as RNA interactome capture, is aiding the investigation of RNA–protein interaction across multiple cell types (357, 358). The efficacy of RNA interactome capture opens the possibility of coupling it with different fractionation strategies to obtain functional maps of interacting molecules, bridging the RNA and protein fields. Harnessing these innovations in a spatial context would unearth new layers of cellular control.

## Conclusion

Coupling -omics with localization studies is still largely in its infancy but is rapidly growing because of advancement of sample preparation strategies and equipment reaching a pinnacle with single-molecule tracking, sequencing, and current MS technology. Not only have subcellular -omics technologies aided our insight into global spatial organization (*e.g.*, HPA Cell Atlas), biological processes (*e.g.*, cell cycle and embryonic development), and pathologies (*e.g.*, cancer biology) but are also emerging in diagnostic applications for patients (10, 40, 76, 161, 166, 314, 341). The hope is that more cell biologists utilize these methods to enrich their own datasets as well as contribute to growing repositories, such as UniProt and the HPA Cell Atlas, with the aim to unearth further understanding of the complex multilayered mechanisms of biological functions and disease (40, 42).

## Conflict of interest

The authors declare no competing interests.
